# Molecular Differences in Invasive Encapsulated Follicular Variant of Papillary Thyroid Carcinoma (IEFVPTC) and Infiltrative Follicular Variant of Papillary Thyroid Carcinoma (IFVPTC): The Role of Extracellular Matrix

**DOI:** 10.3390/biom15121666

**Published:** 2025-11-29

**Authors:** Rebecca Sparavelli, Riccardo Giannini, Laura Boldrini, Beatrice Fuochi, Agnese Proietti, Francesca Signorini, Liborio Torregrossa, Gabriele Materazzi, Clara Ugolini

**Affiliations:** 1 Department of Surgical, Medical, Molecular Pathology and Critical Area, University of Pisa, 56126 Pisa, Italy; 2 Surgery Unit, Department of Surgical, Medical, Molecular Pathology and Critical Area, University Hospital of Pisa, 56126 Pisa, Italy

**Keywords:** PTC, IEFVPTC, IFVPTC, tumor microenvironment, TIMP2, COL1A2

## Abstract

The 2022 WHO classification gives more importance to the integration of morphological and molecular characteristics of tumors, introducing new diagnostic criteria for papillary thyroid carcinoma (PTC). The invasive encapsulated follicular variant of PTC (IEFVPTC) is now considered a separate entity and no longer a subtype of PTC, while the infiltrative follicular variant (IFVPTC) is still considered a PTC subtype. The separation of invasive encapsulated follicular variants from PTCs implies that differential diagnosis between IEFVPTC and IFVPTC can be difficult. We performed a gene expression analysis by NanoString technology on 23 PTCs, divided into 11 IEFVPTCs and 12 IFVPTCs. We focused our attention on the possible role of the tumor microenvironment (TME) and, in particular, the role of the extracellular matrix (ECM). IFVPTC, compared to IEFVPTC, showed a statistically significant downregulation of 2 genes and an upregulation of 45. Among these genes, we focused our attention on *TIMP2* and *COL1A2*, whose high upregulation was statistically significant in IFVPTC. *TIMP2* and *COL1A2* are involved in ECM degradation and synthesis and in collagen biosynthesis and modification. The ECM and collagen alterations in IFVPTC could reflect the different tumor behavior of FVPTCs, allowing the identification of new biomarkers to distinguish IEFVPTC from IFVPTC.

## 1. Introduction

The fifth edition of the World Health Organization (WHO) classification of endocrine tumors was released in 2022. Several novelties have been introduced concerning the nomenclature and histopathological diagnosis of follicular-derived thyroid neoplasms [1]. The 2022 WHO classification places greater emphasis on the integration of morphological and molecular features for tumor characterization and, among its most significant updates, introduces new diagnostic criteria for papillary thyroid carcinoma (PTC) [1]. Among these, the invasive encapsulated follicular variant (IEFVPTC) is now defined as a separate pathological entity, while the infiltrative follicular variant (IFVPTC) continues to be regarded as a subtype of PTC [2].

IEFVPTC is a malignant well-differentiated follicular cell-derived neoplasm that is encapsulated, with a fibrous capsule or well-defined border, and has an exclusive or almost exclusive follicular architecture, nuclear features of PTC, and invasive growth. The molecular profile of IEFVPTC is like that of follicular adenoma and follicular thyroid carcinoma (FTC); *RAS* point mutations, the most frequent somatic alteration detected in IEFVPTC, are present in up to two thirds of cases [3]. In contrast to classic PTC and infiltrative FVPTC, *BRAF* p. V600E mutation is infrequent in strictly defined IEFVPTC [3].

Like FTC, IEFVPTCs also demonstrate a correlation between the extent of invasion and patient prognosis. To better capture this relationship, a diagnosis of IEFVPTC requires the presence of capsular and/or vascular invasion, and the tumor is subclassified into three categories:

1. Minimally invasive—showing capsular invasion only;
2. Encapsulated angioinvasive—exhibiting venous invasion, with or without capsular penetration;
3. Widely invasive—where the tumor grossly invades the surrounding thyroid parenchyma.

Additionally, the mitotic count should be fewer than three per 2 mm^2^, and tumor necrosis should not be present in IEFVPTC [4].

Infiltrative FVPTCs are *BRAF*-driven tumors with an exclusive or almost exclusive follicular architecture, florid nuclear features of PTC, and invasion of the surrounding thyroid parenchyma and lymphatic vessels. After classic PTC, IFVPTC is the second most common histologic subtype of PTC and clinically behaves like classic PTC [4,5]. The separation of invasive encapsulated follicular variants from PTC underscores the need for meticulous evaluation of the tumor capsule and precise reporting of the extent of invasion in the pathological assessment [1]. Differential diagnosis between IEFVPTC and IFVPTC can be difficult and the molecular differences that may explain the distinctive attitude of IEFVPTC and IFVPTC are not completely understood. Recent research has increasingly focused on the contribution of the tumor microenvironment (TME), particularly the extracellular matrix (ECM), to tumorigenesis and cancer progression. During cancer development, the ECM architecture becomes disrupted, accompanied by alterations in its biochemical composition and mechanical properties [6]. Changes in the ECM—including its protein composition, activity, and cross-linking—lead to altered cell signaling [6]. The dynamic equilibrium between ECM synthesis and degradation, coordinated by ECM-remodeling cells, underlies tensional homeostasis and defines the mechanical characteristics of individual organs, including elasticity, compressive resistance, and tensile strength [7]. In most cancers, ECM remodeling involves greater collagen production and activation of remodeling enzymes such as matrix metalloproteinases (MMPs) [8]. A growing body of evidence indicates that tumor progression and invasiveness are largely mediated by ECM-remodeling and matrix-degrading enzymes, which profoundly reshape the architecture and composition of the tumor microenvironment (TME) [9]. As a result, ECM abnormalities have emerged as a hallmark of aggressive behavior and unfavorable prognosis in multiple malignancies, such as breast, renal, and head and neck cancers [10].

Therefore, this study aimed to analyze gene expression profiles of >700 genes involved in the four stages of fibrosis (initiation, inflammation, proliferation, and modification) using the NanoString nCounter Fibrosis Profiling Panel and the Fibrosis Profiling Advanced Analysis Module to further investigate the role of the tumor microenvironment and extracellular matrix in FVPTCs and identify possible new biomarkers in distinguishing between IEFVPTCs and IFVPTCs.

## 2. Materials and Methods

### 2.1. Sample Selection

A consecutive series of 23 patients affected by FVPTC, diagnosed between 2017 and 2023 at the Unit of Surgical Pathology of the University Hospital of Pisa, were included in this study. Patients underwent total thyroidectomy or lobectomy at the Endocrine Surgery Unit of the University Hospital of Pisa and, whenever appropriate, lymph node dissection and radioiodine treatment according to the American Thyroid Association (ATA) guidelines were performed [11]. For each case, glass slides of the primary tumor samples, which were formalin-fixed paraffin-embedded (FFPE), were retrieved and re-evaluated independently by two pathologists (F.S. and C.U.) according to the 2022 WHO criteria [4]. FVPTCs were divided into two groups: 11 invasive encapsulated FVPTCs (IEFVPTCs, with capsular and/or vascular invasion) and 12 infiltrative FVPTCs (IFVPTCs, with parenchymal infiltration with or without vascular invasion). Tumors smaller than 3 mm or displaying features of aggressive variants—such as hobnail, tall cell, columnar, or solid/trabecular patterns—were excluded from the study. For each patient, clinicopathological characteristics were evaluated, including age at diagnosis, sex, tumor size, capsular infiltration, vascular invasion, parenchymal infiltration, extrathyroidal extension (ETE), multifocality, bilaterality, lymph node metastasis, and the presence of associated goiter or thyroiditis.

For histological assessment, the most representative paraffin block from each case, containing both tumor and tumor microenvironment (TME) areas, was selected for analysis. The study was conducted anonymously and in accordance with the ethical principles of the Declaration of Helsinki and approved by CEAVNO Protocol no. 9989, 20 February 2019.

### 2.2. Nucleic Acid Extraction and Purification

For each sample, four unstained sections (5 µm thick) were used for RNA extraction. The sections were deparaffinized with xylene and rehydrated through a graded ethanol series. Manual microdissection was performed to enrich for tumor cells and tumor microenvironment (TME) components. Total RNA was isolated using the RNeasy FFPE Kit (Qiagen, Hilden, Germany), following the manufacturer’s instructions, and eluted in 18 µL of RNase-free water. The RNA concentration and purity were assessed using a spectrophotometer (Xpose, Trinean, Gentbrugge, Belgium).

### 2.3. Differential Gene Expression Analysis

Analysis of the expression profiles of >700 genes involved in the four stage of fibrosis (initiation, inflammation, proliferation, and modification) was performed by the NanoString nCounter Fibrosis Profiling Panel (NanoString Technologies, Seattle, WA, USA). In detail, 100 ng of RNA from each sample was hybridized with the nCounter Fibrosis Profiling Panel (GX Assay) CodeSet. All procedures related to mRNA quantification—including sample preparation, hybridization, detection, and scanning—were performed according to the manufacturer’s instructions. Counts were normalized following the standard protocol. Raw NanoString counts for each mRNA were first subjected to technical normalization using the positive control probe sets, followed by biological normalization using the 40 reference genes provided in the CodeSet. The normalized data were log2 transformed and then used as input for differential expression analysis. The Fibrosis Profiling Advanced Analysis Module (NanoString Technologies) was used to conduct the statistical analysis of data obtained by the nCounter panel analysis. For biological (sample content) normalization, the panel included 10 candidate housekeeping genes. Housekeeping selection and normalization were carried out with the nSolver Advanced Analysis “housekeeping” module, which implements a geNorm-based algorithm to identify the most stable reference genes across all samples. Based on this analysis, 8 genes (*GUSB*, *PGK1*, *ARMH3*, *ACAD9*, *NUBP1*, *RPLP0*, *MTMR14*, and *PPIA*) with the lowest variability were retained and used for normalization, whereas *NOL7* and *CNOT10* were excluded due to higher standard deviation after normalization. For each sample, the geometric mean of the counts of the selected housekeeping genes was calculated and used as a normalization factor for all target genes. The resulting normalized expression values were used for all downstream analyses. The analysis module grouped the genes into functional immune-related categories. To investigate the differential expression between the category of samples in this study, the main covariate considered was the diagnostic classification criteria: IEFVPTC or IFFVPTC. Large number of genes in the CodeSet made the use of raw *p* values problematic. In particular, we considered differentially expressed genes with a significant *p* value without and with Benjamini–Yekutieli correction (BY); this particular type of statistical correction was used for adjusting the *p* value. In conclusion, genes with a fold change > 0.5 and with *p*-value < 0.05 were considered differentially expressed. Furthermore, differentially expressed genes with BY-adjusted *p*-value < 0.05 were reported and considered as the most statistically significant differentially expressed genes.

## 3. Results

### 3.1. Sample Features

A series of 23 patients affected by FVPTC were examined, of which 11 were diagnosed as encapsulated FVPTCs (IEFVPTCs, with capsular and/or vascular invasion) and 12 as infiltrative FVPTCs (IFVPTCs, with parenchyma infiltration with or without vascular invasion), according to the 2022 WHO criteria.

The mean age was 52.5 years old (range of 28–83 years old). A total of 61.5% of patients were female. The mean size of the neoplasms was 1.9 cm (range of 0.3–5 cm). Overall, 27% of patients experienced local recurrences while no patients developed distant recurrences or metastases. All data are summarized in Table 1.

### 3.2. Differential Gene Expression Analysis

The housekeeping genes selected for the normalization of the experiment presented a steady expression level in all the studied samples. None of the samples was excluded after data normalization.

**Figure 1:**
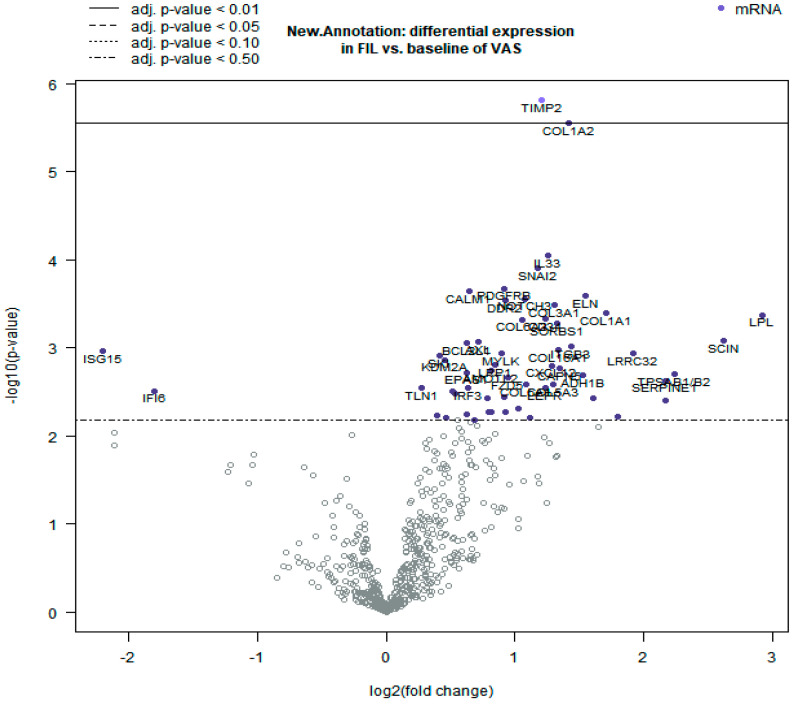
Gene expression data. Volcano plots displaying each gene’s −log10 (*p*-value) and log-two-fold change. Highly statistically significant genes fall at the top of the plot above the horizontal lines, and highly differentially expressed genes fall to either side. Horizontal lines indicate various *p*-value thresholds. Genes are colored if the resulting *p*-value is below the given *p*-value threshold.

A comparison between IFVPTC and IEFVPTC was performed. Differentiated genes are visualized in the specific volcano plot, a graph displaying each gene’s log10 BY-*p* value and log2 fold change for the specific categories of samples (Figure 1). As we can see in Figure 1, IEFVPTC, compared to IFVPTC, showed a statistically significant deregulation of 47 genes. In particular, we observe a downregulation of 2 genes and an upregulation of 45. Two of these genes, *TIMP2* and *COL1A2*, underwent statistically significant upregulation after application of BY correction (BY *p*-value < 0.01). The complete lists of deregulated genes are reported in Table S1.

**Figure A1:**
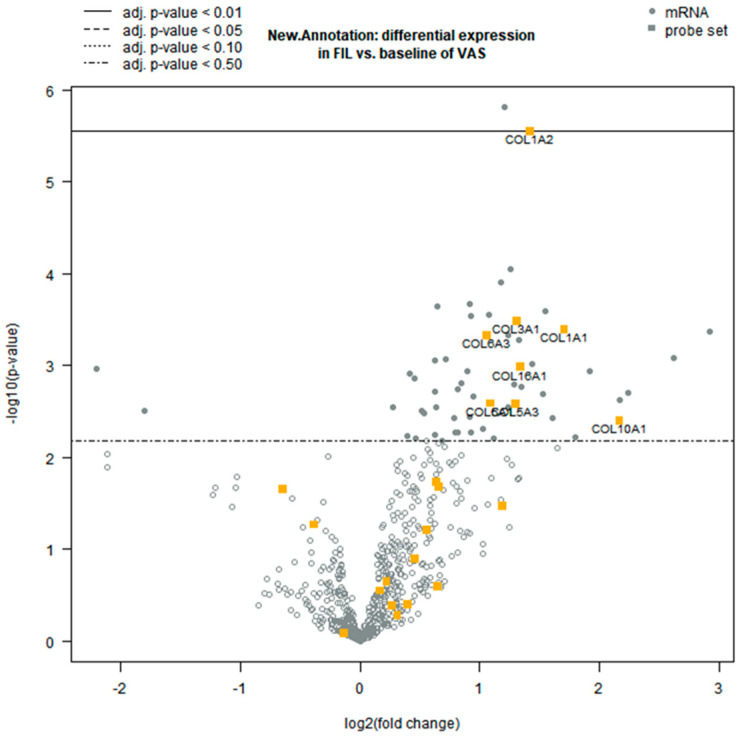
Collagen biosynthesis and modification. Global significant score. Volcano plots displaying each gene’s −log10 (*p*-value) and log-two-fold change. Differentially expressed genes, revealed by differential analysis and involved in collagen biosynthesis and modification, are displayed in yellow.

**Figure A2:**
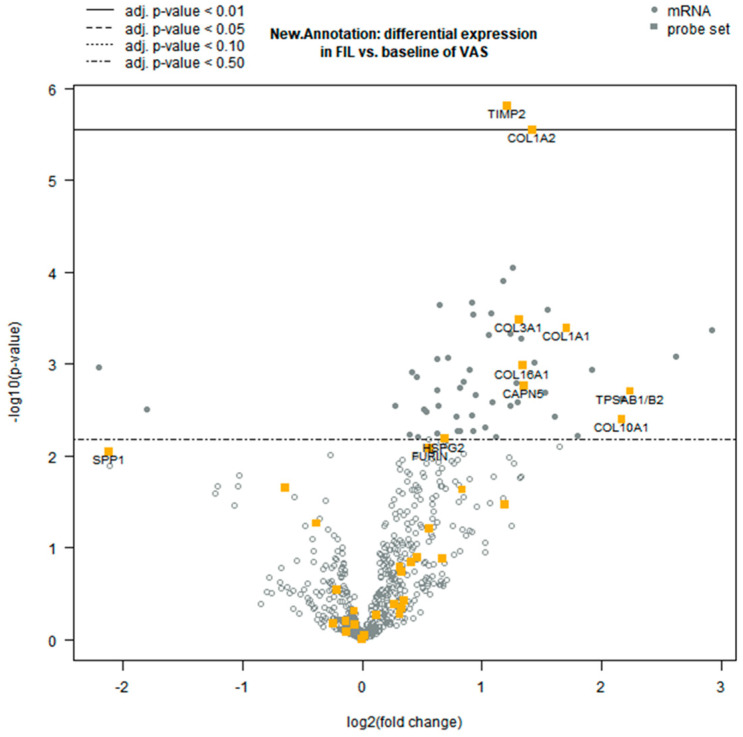
ECM degradation. Global significant score. Volcano plots displaying each gene’s −log10 (*p*-value) and log-two-fold change. Differentially expressed genes, revealed by differential analysis and involved in ECM degradation, are displayed in yellow.

**Figure A3:**
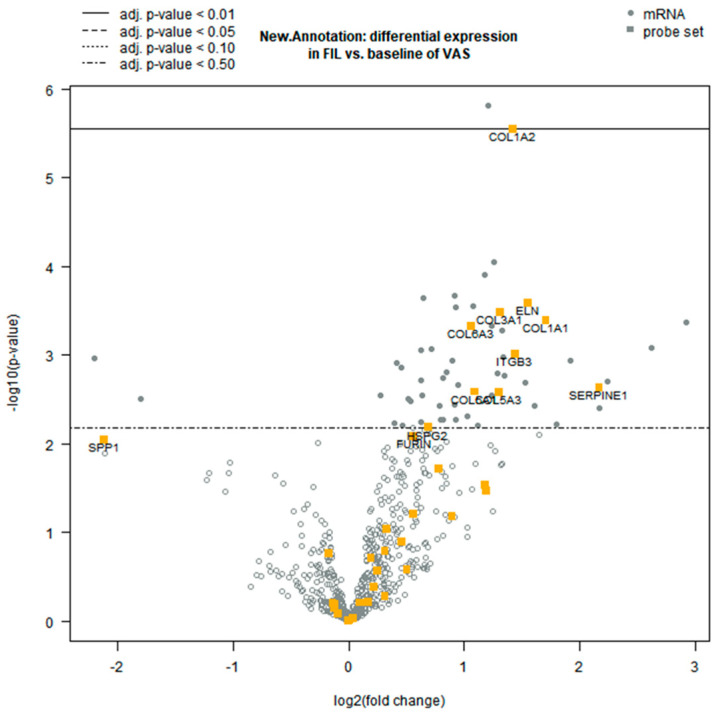
ECM synthesis. Global significant score. Volcano plots displaying each gene’s −log10 (*p*-value) and log-two-fold change. Differentially expressed genes, revealed by differential analysis and involved in ECM synthesis, are displayed in yellow.

In the analysis of global significance score, the upregulated genes, such as *COL1A2*, *TIMP2*, *COL3A1*, *COL6A3*, *COL16A1* etc., were included in functional group of genes involved in collagen biosynthesis and modification (Figure A1), ECM degradation (Figure A2), and ECM synthesis (Figure A3), showed in Appendix A.

### 3.3. Functional Clustering Analysis

**Figure 2:**
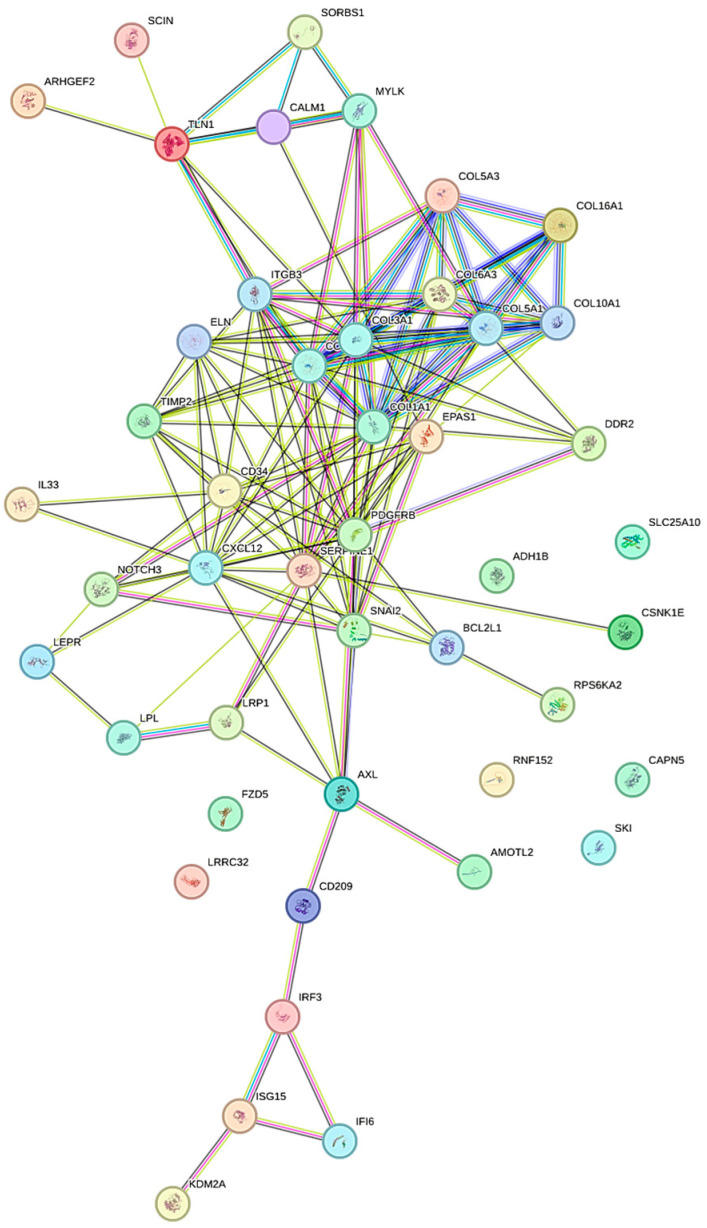
In “silico” analysis based on protein–protein interactions, both physical and functional associations, of IFVPTC and IEFVPTC, retrieved from STRING. The edges represent specific and statistically significant protein–protein associations, which do not necessarily imply direct physical interactions. Distinct colors denote known or predicted interaction types.

Based on the interactions retrieved from STRING, a protein network for our categories is constructed. The IEFVPTC network, compared to IEFVPTC, showed 47 proteins and 141 edges, as we can see in Figure 2. For the clustering analysis, the non-parametric “Aggregate Fold Change” method was employed. This approach computes, for each gene set, the mean value of all measurements provided for its constituent genes. *p*-values were adjusted using the false discovery rate (FDR), which provides an estimate of the expected proportion of false-positive findings. In this study, for the IEFVPTC network, among the 47 differentially expressed genes, 15 were enriched in pathways involved in blood vessel development, with 9 in extracellular matrix structural constituents and platelet-derived growth factor bindings (*p*-value FDR: 6.63 × 10^−9^, 7.60 × 10^−8^, 3.86 × 10^−7^).

## 4. Discussion

Thyroid carcinoma is a heterogeneous disease, exhibiting distinct histopathological and biological characteristics across its various subtypes [1]. It occurs more frequently in women and represents the most common neoplasm of the endocrine system, accounting for approximately 4% of all malignant tumors [12]. Most thyroid cancers are classified as differentiated tumors, primarily comprising papillary thyroid carcinoma (PTC) and follicular thyroid carcinoma (FTC), which represent about 80–90% and 10–20% of differentiated thyroid neoplasms, respectively [10].

In recent years, the 2022 WHO classification has introduced significant updates that have led to substantial revisions in the diagnostic criteria for the follicular variant of PTC [1]. The invasive encapsulated follicular variant of PTC (IEFVPTC) is now recognized as a distinct pathological entity and is no longer classified as a subtype of PTC, whereas the infiltrative follicular variant (IFVPTC) remains categorized within the spectrum of PTC subtypes [1,2]. For prognostic risk stratification, IEFVPTC should be further subclassified into minimally invasive (characterized by capsular invasion only), encapsulated angioinvasive, and widely invasive forms—the latter defined by complete or near-complete capsule destruction and/or gross extrathyroidal invasion. Although PTCs are generally associated with excellent long-term survival, IEFVPTC may exhibit distant metastatic spread, often bypassing regional lymph nodes, in a manner similar to that observed in FTC [2]. The factors involved are not well known, and we focused our attention on the possible role of the tumor microenvironment (TME) and, in particular, the role of the extracellular matrix (ECM), in the tumor process.

Recent studies have examined the roles of key proteins involved in the regulation of the extracellular matrix, identifying metalloproteinases and their endogenous inhibitors as central mediators of the processes governing matrix synthesis and degradation [13,14,15]. Matrix metalloproteinases (MMPs) constitute a family of proteolytic enzymes synthesized and secreted by various cell types—including macrophages, neutrophils, lymphocytes, and fibroblasts—and are principally involved in the degradation of the extracellular matrix (ECM). Under physiological conditions, active MMPs degrade ECM components and modulate the bioavailability of growth factors, cytokines, and chemokines, thereby regulating tissue remodeling processes essential for immune function, cellular proliferation, angiogenesis, morphogenesis, skeletal development, and tissue repair [13].

In pathological contexts, MMPs contribute to tumor invasion and metastasis by facilitating the breakdown of structural barriers and promoting tumor cell dissemination [14]. Cross-talk between MMPs and upstream oncogenic pathways can modulate MMP activity, highlighting opportunities for combined therapeutic strategies to more effectively counter PTC aggressiveness [15]. Their activity is tightly regulated at multiple levels, including transcriptional control and activation of latent proenzymes, and is counterbalanced by endogenous tissue inhibitors of metalloproteinases (TIMPs) [13,16]. The TIMP family consists of four members—tissue inhibitor of metalloproteinase-1 (TIMP1), tissue inhibitor of metalloproteinase-2 (TIMP2), tissue inhibitor of metalloproteinase-3 (TIMP3), and tissue inhibitor of metalloproteinase-4 (TIMP4)—which inhibit MMP proteolytic activity [17]. TIMP-2 regulates proteolysis of the ECM and cell-surface proteins through inhibition of MMP activity [18]. Interestingly, TIMP-2 is unique in that beyond inhibiting MMP activity, it selectively interacts with membrane-type MMPs to facilitate the cell-surface activation of pro-MMP-2 [19,20]. Accordingly, its role in regulating the thyroid tumor extracellular matrix warrants further investigation. Although individual affinities vary, all TIMPs are generally regarded as endogenous inhibitors of MMPs [21]. TIMP-1 displays the highest affinity for MMP-9, which plays a significant role in immune cell function and fibrosis in cardiovascular disease [22]. TIMP-3 exhibits strong interactions with low-density lipoprotein receptor-related protein 1 (LRP-1), several ADAM family proteases (including ADAM10), the angiotensin II type-2 receptor (AT2R), MMP-9, and MMP-13 [23]. TIMP-4 serves as a key regulator of MMP-9 and protease-activated receptor-1 (PAR-1), thereby influencing cell polarity [24,25].

TIMP-2 forms high-affinity complexes with a broad range of MMPs, including MMP-1, MMP-2, MMP-3, MMP-7, MMP-8, MMP-9, MMP-10, MMP-13, MMP-14, MMP-15, MMP-16, and MMP-19 [12]. Among these, MMP-2 is of particular interest as an anticancer therapeutic target due to its high expression across multiple malignancies, including breast, cervical, bladder, gastric, and lung cancers [12]. There remains considerable debate regarding the role of TIMP2 in cancer. While some studies indicate that TIMP2 exerts tumor-suppressive functions, others associate it with enhanced tumor cell survival and proliferation. These divergent observations are thought to arise from the context-dependent nature of TIMP2’s interaction with MMP14 [26].

Type I collagen, which is abundant in most connective and embryonic tissues, represents a major constituent of the collagen family and serves as a critical structural element of the extracellular matrix (ECM). It is conventionally composed of two collagen type I alpha-1 (COL1A1) chains and one collagen type I alpha-2 (COL1A2) chain [27]. Recent studies have reported abnormal expression of *COL1A1* and *COL1A2* in osteogenesis, osteoporosis, various bone disorders, and multiple cancers [10,11,12,13,14,15,16,17,18,19,20,21,22,23,24,25,26,27]. The *COL1A2* gene has been extensively investigated in experimental models aimed at elucidating the molecular mechanisms underlying type I collagen biosynthesis. Increasing evidence suggests that dysregulated type I collagen expression contributes to the carcinogenesis of several human cancers, including gastric and pancreatic malignancies [28].

However, the expression patterns of *COL1A1* and *COL1A2* in malignant tumors remain a matter of debate. On one hand, frequent promoter methylation of *COL1A1* has been observed in renal cell carcinoma and hepatocellular carcinoma, and *COL1A2* downregulation has been reported in melanoma, head and neck cancer, and bladder cancer [27]. On the other hand, elevated mRNA levels of both *COL1A1* and *COL1A2* have been documented in colorectal, liver, ovarian, and pancreatic cancers, as well as in medulloblastoma [27,28]. Huang et al. demonstrated that the extracellular matrix could influence the degree of malignancy and progression of PTC. Their findings indicate that *COL1A1* holds significant clinical value as a diagnostic and prognostic biomarker, with potential utility in assessing disease-free survival and recurrence in PTC [10].

A key example of the importance of ECM regulation within the TME is the epithelial–mesenchymal transition (EMT). EMT is a morphological transition driven by multiple factors, including a decrease in cell–cell adhesion and an enhanced degradation of extracellular matrix components that facilitate cell migration [29]. Evidence increasingly shows that EMT activation is closely linked to thyroid carcinoma progression, including extrathyroidal extension, distant metastasis, and heightened cancer cell stemness; however, the clinical significance of this association remains to be fully elucidated [30]. Elucidating the regulation of the ECM and the proteins involved may help clarify the mechanisms underlying this process, which is closely linked to enhanced invasive and metastatic potential [31]. MMPs facilitate EMT by cleaving, shedding, or degrading a broad range of cell-surface molecules [32,33]. For example, Li Z. et al. said that elevated MMP-9 levels are closely associated with enhanced invasion, migration, metastasis, and apoptosis of thyroid cancer cells. Conversely, inhibition of MMP-9 suppresses EMT, thereby slowing tumor progression, prolonging survival [34].

We focused our attention on FVPTCs, particularly on encapsulated FVPTCs (IEFVPTCs) and infiltrative FVPTCs (IFVPTCs), according to the 2022 WHO criteria. We conducted an analysis on the gene expression profiles of >700 genes involved in the four stages of fibrosis (initiation, inflammation, proliferation, and modification) using the NanoString nCounter Fibrosis Profiling Panel. IFVPTC, compared to IEFVPTC, showed a statistically significant deregulation of 47 genes; in particular, 2 genes downregulated and 45 upregulated. Among these genes, we focused our attention on *TIMP2* and *COL1A2*, which were identified as significantly deregulated following multiple testing correction using the Benjamini–Yekutieli method. TIMP2 and COL1A2 are involved in ECM degradation and synthesis by remodeling collagen; TIMP2 regulates ECM proteolysis through inhibition of metalloproteinase activity as MMP2, but also metalloproteinases activation as MMP-2 and MMP-14, promoting stromal infiltration and possible development of metastasis [21,26]. *COL1A2* plays a critical role in collagen synthesis and, when dysregulated, may contribute to enhanced cell proliferation and metastatic potential [35]. Thus, the extracellular matrix alterations associated with the upregulation of *TIMP2* and *COL1A2* in IFVPTC may help explain the distinct biological behavior observed between IEFVPTC and IFVPTC.

In general, IEFVPTCs exhibit a more favorable prognosis than IFVPTCs, and further histological subclassification of the follicular variant of PTC is unlikely to substantially alter clinical management strategies [4,36]. Nevertheless, distinguishing IEFVPTCs from invasive PTCs requires meticulous evaluation of the tumor capsule and precise documentation of the extent of tumor invasiveness in the histopathological report [4]. This focus is particularly important, as patients with widely invasive encapsulated follicular variants are at a higher risk of developing distant metastases and may therefore benefit from more aggressive therapeutic approaches [4]. Widely invasive IEFVPTCs may also pose diagnostic challenges in differentiating them from IFVPTCs, especially when the tumor capsule is inconspicuous. Consequently, the identification of novel biomarkers that facilitate reliable distinction between these two tumor forms is essential. As demonstrated in our study, the discovery of new molecular biomarkers may emerge from a deeper examination of the TME, particularly through the analysis of the four stages of fibrosis. The observed upregulation of *TIMP2* and *COL1A2* in IFVPTCs, compared with IEFVPTCs, underscores distinct differences in extracellular matrix composition and collagen-related features between these tumors. Understanding the role of ECM in the TME could reflect and justify the molecular alterations related to the different tumor behavior of IEFVPTCs and IFVPTCs. Despite the number of samples selected for this study possibly appearing limited, the robustness of the NanoString technology used for gene expression analysis is very high. The platform includes numerous internal controls, including sample quality controls and normalization with housekeeping genes, ensuring reliable and accurate measurements even with a reduced sample size. Furthermore, the primary objective of this study is to gather preliminary data to inform future research hypotheses rather than to establish definitive conclusions. Moving forward, additional studies involving a larger cohort of cases will be necessary to elucidate the role of the extracellular matrix more clearly in distinguishing these tumor types.

## 5. Conclusions

The 2022 WHO classification introduced several updates regarding the nomenclature and histopathological diagnosis of follicular-derived thyroid neoplasms. Notably, the invasive encapsulated follicular variant of papillary thyroid carcinoma (IEFVPTC) is now recognized as a distinct entity, whereas the infiltrative follicular variant (IFVPTC) continues to be classified as a subtype of papillary thyroid carcinoma. Differential diagnosis between IEFVPTC and IFVPTC can be challenging, particularly when the tumor capsule is inconspicuous. Therefore, there is a clear need to identify novel biomarkers capable of facilitating a reliable distinction between these two tumor types. The evaluation of tumor microenvironment by deepening the four stages of the fibrosis process could help to identify molecular biomarkers involved in the different behavior of these tumors. As evidenced by our study, the upregulation of TIMP2 and COL1A2 in IFVPTC, compared to IEFVPTC, could reflect and justify the different features of these malignancies in order to improve the histopathological diagnosis and the patients’ management.

## Figures and Tables

**Table 1:** Patient characteristics. Abbreviations: IEFVPTC—Invasive encapsulated follicular variant of PTC; IFVPTC—Infiltrative follicular variants of PTC; sx—left; dx—right; inv—invasion; inf—infiltration; CN—capsular neoplasm; IP—parenchyma infiltration; CT—thyroid capsular; TNM—Tumor node metastasis staging system.

Sample ID	IEFVPTC/IFVPTC	Tumor Location	Size	Type of inv/inf	Embolism	nr Embolisms	TNM
VAS1	IEFVPTC	lobe sx	3.8 cm	CN	N	0	pT2(m)N0Mx
VAS6	IEFVPTC	lobe sx	0.7 cm	CN, foc IP	Y	1	pT1a(m)NxMx
VAS8	IEFVPTC	lobe dx	5 cm	CN	Y	1	pT3aNxMx
VAS2	IEFVPTC	lobe sx	1.5 cm	CN	N	0	pT1bNxMx
VAS3	IEFVPTC	lobe dx	0.5 cm	CN	N	0	pT2(m)N0Mx
VAS4	IEFVPTC	isthmus	3.5 cm	CN, IP, CT	N	0	pT2NxMx
VAS5	IEFVPTC	lobe dx	4.5 cm	CN	Y	4	pT3aNxMx
VAS7	IEFVPTC	lobe sx	3 cm	CN	N	0	pT2NxMx
VAS9	IEFVPTC	lobe dx	1.8 cm	CN, foc IP	Y	<4	pT1b(m)NxMx
VAS10	IEFVPTC	lobe sx	2.2 cm	CN	N	0	pT2NxMx
VAS11	IEFVPTC	lobe sx	2.7 cm	CN	Y	>4	pT2(m)N1aMx
VAS12	IEFVPTC	lobe dx	1.7 cm	CN	N	0	pT1bNxMx
VAS13	IEFVPTC	lobe sx	2.7 cm	CN	N	0	pT2NxMx
FIL2	IFVPTC	lobe dx	1.4 cm	IP, TL	Y	>4	pT1b(m)N1aMx
FIL1	IFVPTC	lobe sx	0.7 cm	IP	N	0	pT1aNxMx
FIL3	IFVPTC	lobe dx	2.5 cm	IP	N	0	pT2(m)NxMx
FIL5	IFVPTC	lobe dx	0.8 cm	TL	Y	>4	pT1b(m)N1bMx
FIL6	IFVPTC	lobe sx	3.6 cm	IP, TL	Y	>4	pT2(m)N1bMx
FIL7	IFVPTC	lobe dx	1.5 cm	IP	N	0	pT1bN0Mx
FIL8	IFVPTC	lobe dx	1 cm	IP, CT	N	0	pT1a(m)N1aMx
FIL4	IFVPTC	lobe dx	1 cm	TL	Y	4	pT1a(m)N1bMx
FIL9	IFVPTC	lobe sx	0.3 cm	IP	N	0	pT1aNxMx
FIL10	IFVPTC	lobe sx	1.5 cm	IP, CT	N	0	pT1bNxMx
FIL11	IFVPTC	lobe dx	0.6 cm	IP, TL	Y	<4	pT1a(m)N1bMx
FIL12	IFVPTC	lobe dx	0.6 cm	IP	N	0	pT1aNxMx
FIL13	IFVPTC	lobe dx	0.4 cm	IP	N	0	pT1aNxMx

## Data Availability

The raw data supporting the conclusions of this article will be made available by the authors upon request.

## Supplementary Materials

The following supporting information can be downloaded at https://www.mdpi.com/article/10.3390/biom15121666/s1, Table S1: Complete lists of deregulated genes between IEFVPTC and IFVPTC.

## Abbreviations

The following abbreviations are used in this manuscript:

BY	Benjamini–Yekutieli
COL1A1	Collagen type I alpha 1
COL1A2	Collagen type I alpha 2
ECM	Extracellular matrix
EMT	Epithelial–mesenchymal transition
ETE	Extra-thyroid extension
FFPE	Formalin-fixed paraffin-embedded
FTC	Follicular thyroid cancer
IEFVPTC	Invasive encapsulated follicular variant of papillary thyroid cancer
IFVPTC	Infiltrative follicular variant of papillary thyroid cancer
MMPs	Metalloproteinases
PTC	Papillary thyroid cancer
TME	Tumor microenvironment
TIMPs	Tissue inhibitors of metalloproteinases
WHO	World Health Organization
